# Development and Preliminary Assessment of a Mortality Risk Score in Patients with Coronary Artery Disease Receiving Dual Antiplatelet Therapy After Percutaneous Coronary Intervention

**DOI:** 10.3390/clinpract16080149

**Published:** 2026-08-14

**Authors:** Friba Nurmukhammad, Sholpan Zhangelova, Akhmetzhan Sugraliyev, Alexander Arutyunov, Yermagambet Kuatbayev, Zhanetta Mukanova, Dina Kapsultanova

**Affiliations:** 1 Faculty of Postgraduate Medical Education, Khoja Akhmet Yassawi International Kazakh-Turkish University, Turkestan 161200, Kazakhstan; friba_93@mail.ru; 2 Department of Internal Diseases, School of General Medicine, Asfendiyarov Kazakh National Medical University, Almaty 050012, Kazakhstan; zhangelova.s@kaznmu.kz (S.Z.); zhanetta2000@mail.ru (Z.M.); 3 City Cardiology Center, Almaty 050012, Kazakhstan; y.kuat2025@gmail.com

**Keywords:** coronary artery disease, dual antiplatelet therapy, percutaneous coronary intervention, in-hospital mortality, exploratory risk score, clinical risk stratification, clopidogrel

## Abstract

**Background**: Patients with coronary artery disease (CAD) receiving dual antiplatelet therapy (DAPT) after percutaneous coronary intervention (PCI) remain at risk of early adverse outcomes, including in-hospital mortality. Simple risk stratification based on routinely available variables may help identify higher-risk patients, but a limited number of outcome events constrains robust prediction-model development and validation. **Aim**: This exploratory study aimed to derive a preliminary, interpretable clinical score based on routinely available variables for risk stratification of all-cause in-hospital mortality in CAD patients receiving DAPT after PCI. In the clopidogrel-dominant practice setting of the participating centers, the score was conceived as a hypothesis-generating risk-enrichment framework rather than a validated treatment-selection tool or a surrogate measure of platelet reactivity. **Methods**: We analyzed a retrospective cohort of 1600 adults with CAD admitted between 2022 and 2024; 36 in-hospital deaths occurred. Twenty demographic, clinical, laboratory, and instrumental variables were evaluated. The primary outcome was all-cause in-hospital mortality during the index hospitalization. For exploratory score derivation, the dataset was randomly divided into a derivation subset (75%; *n* = 1200) and a hold-out assessment subset (25%; *n* = 400). Predictors were explored using univariable and multivariable logistic regression with stepwise selection. Continuous variables were categorized using Weight of Evidence binning, and an integer point score was derived. Performance was summarized using ROC analysis, AUC, sensitivity, specificity, and accuracy. Given the small number of deaths and the data-driven modelling workflow, all performance estimates were considered preliminary rather than definitive internal validation. **Results**: The exploratory six-variable score included age ≥ 57 years, estimated glomerular filtration rate < 45 mL/min/1.73 m^2^, body mass index ≥ 25 kg/m^2^, troponin I ≥ 100, prior myocardial infarction, and current smoking. In the derivation subset, each additional point was associated with higher odds of mortality (OR 1.39; 95% CI 1.29–1.51; *p* < 0.001), and the AUC was 0.654. A Youden-index threshold of approximately 6 points yielded sensitivity of 0.41, specificity of 0.80, and accuracy of 0.72. In the hold-out assessment subset, sensitivity was 0.53, specificity was 0.70, accuracy was 0.70, and AUC was 0.61. These estimates indicate modest discrimination and should be interpreted cautiously because only 36 outcome events were available. **Conclusions**: This exploratory clinical score showed modest discrimination for all-cause in-hospital mortality and should be regarded as a preliminary, hypothesis-generating risk-stratification approach. It is not sufficiently validated for routine prognostic classification, platelet-reactivity triage, or antiplatelet treatment selection. Model redevelopment using event-efficient methods, resampling-based internal validation, and subsequent external validation are required before clinical implementation.

## 1. Introduction

Acute coronary syndrome (ACS) remains one of the major causes of cardiovascular morbidity and mortality worldwide. In most cases, ACS develops after rupture or erosion of an unstable coronary atherosclerotic plaque, followed by platelet activation, thrombus formation, and acute myocardial ischemia. Platelet activation is a central mechanism in this process: after endothelial injury, platelets release adenosine diphosphate (ADP), which stimulates P2Y12 receptors, amplifies platelet aggregation, and promotes coronary thrombosis [1].

Percutaneous coronary intervention (PCI) combined with dual antiplatelet therapy (DAPT) is a standard strategy for reducing recurrent ischemic events in patients with ACS and coronary artery disease (CAD). However, despite guideline-based antiplatelet therapy, a proportion of patients continue to experience adverse cardiovascular outcomes, including recurrent myocardial infarction, stent thrombosis, stroke, and death. One of the mechanisms underlying this residual risk is high residual platelet reactivity (HRPR), also referred to as high on-treatment platelet reactivity (HTPR), which reflects insufficient platelet inhibition during antiplatelet therapy [1,2].

The clinical relevance of HRPR has been supported by multiple studies. In patients with ACS undergoing PCI and receiving DAPT, HTPR has been associated with an increased risk of major adverse cardiovascular events (MACE), all-cause or cardiac mortality, recurrent myocardial infarction, in-stent restenosis, and stroke. These associations may be explained by persistent platelet aggregation, increased thrombus formation, enhanced activation of coagulation pathways, and insufficient suppression of platelet-mediated inflammation [1,2,3].

Response to clopidogrel is characterized by substantial interindividual variability. Some patients treated with clopidogrel after PCI demonstrate inadequate platelet inhibition and remain at increased risk of recurrent atherothrombotic events. In patients with acute myocardial infarction, elevated platelet reactivity has shown prognostic value for long-term outcomes after coronary angiography and PCI, including recurrent ischemic events and adverse cardiovascular prognosis [2,3].

Platelet reactivity is also a dynamic phenomenon. It may vary during ischemia, reperfusion, PCI, and the early post-procedural period. Previous studies have shown that platelet activity may increase during myocardial ischemia and reperfusion and may be associated with angiographic success, ST-segment resolution, thrombus burden, recovery of left ventricular function, and short- and mid-term clinical outcomes after primary PCI [3,4].

Importantly, platelet reactivity should not be interpreted in isolation. The risk of ischemic complications after PCI is influenced by multiple clinical and laboratory factors, including age, diabetes mellitus, chronic kidney disease, body mass index, inflammatory status, myocardial injury markers, ACS presentation, and timing after PCI. Several of these factors are also associated with impaired response to antiplatelet therapy and may contribute to HRPR [3,4].

Because post-PCI mortality risk is multifactorial and specialized platelet testing is not routinely available in many settings, routinely collected clinical and laboratory variables may be useful for exploratory risk enrichment. However, any score derived from a cohort with few mortality events should be considered preliminary and should not be interpreted as a surrogate measure of platelet reactivity or as an autonomous treatment-selection tool.

This question is particularly relevant in the local clinical context of the Republic of Kazakhstan. During the 2022–2024 study period, clopidogrel-based DAPT was the default strategy at the participating centers, reflecting local prescribing practice, resource stewardship, and drug availability, whereas ticagrelor was used selectively in patients considered to have higher ischemic risk. This was an institutional practice pattern rather than a national restriction: both clopidogrel and ticagrelor were included in the Kazakhstan outpatient benefit list for ischemic heart disease [5]. Contemporary ESC guidance supports the use of more potent P2Y12 inhibition in appropriately selected ACS patients with high ischemic risk, while taking bleeding risk into account [6]. Accordingly, the exploratory score evaluated here is intended only to identify a potentially higher-risk clinical phenotype that may warrant broader reassessment of secondary prevention. The present observational data do not establish that ticagrelor improves outcomes in patients with a high score, and any escalation should remain an individualized clinical decision.

The objective of this study was to explore the derivation of a simple point-based score for all-cause in-hospital mortality risk stratification in patients with CAD receiving DAPT after PCI using routinely collected clinical and laboratory variables, and to obtain a preliminary estimate of its performance in a hold-out subset. The study was not designed to establish a definitive validated prediction model or to predict high on-treatment platelet reactivity.

## 2. Materials and Methods

### 2.1. Study Design and Population

This retrospective cohort study included medical records from adult patients residing in Almaty and the Almaty Region of the Republic of Kazakhstan. Patients were admitted on an emergency or elective basis between 1 January 2022 and 31 December 2024 with a preliminary diagnosis of CAD and underwent coronary angiography during hospitalization.

**Figure 1:**
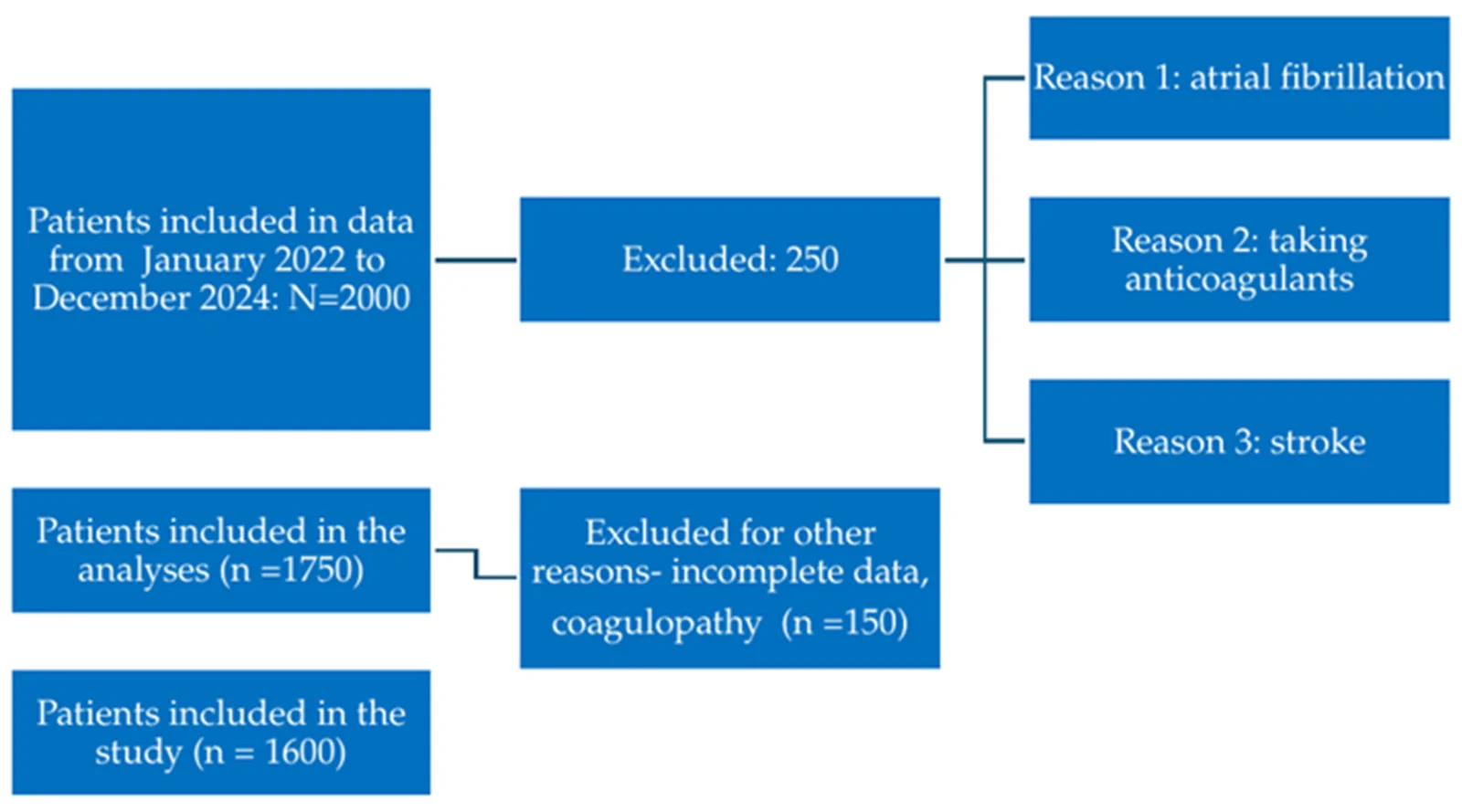
Patient selection flowchart.

Initially, 2000 patient records were reviewed. We excluded 250 patients because of atrial fibrillation, anticoagulant therapy, or previous stroke, and 150 additional records because of incomplete data or coagulopathy. The final analytical cohort included 1600 patients aged ≥ 18 years (Figure 1).

### 2.2. Clinical and Laboratory Data Collection

Baseline demographic, clinical, laboratory, and instrumental data were extracted from electronic medical records. The following 20 variables were evaluated: sex, age, estimated glomerular filtration rate (eGFR), body mass index (BMI), C-reactive protein (CRP), total cholesterol, low-density lipoprotein cholesterol (LDL-C), triglycerides, fibrinogen, D-dimer, troponin I, hemoglobin, type 2 diabetes mellitus, impaired glucose tolerance, chronic obstructive pulmonary disease (COPD), smoking status, history of COVID-19 infection, prior myocardial infarction, coronary vessel disease, and left ventricular ejection fraction.

The endpoint was binary mortality status recorded in the dataset and coded as survived or deceased. Because time-to-event information was not available, survival analysis methods were not applied.

The primary outcome was all-cause in-hospital mortality during the index hospitalization. Post-discharge follow-up was not available, and 30-day, longer-term, or cause-specific cardiovascular mortality could not be assessed. Current smoking was defined as active tobacco use documented at admission by patient self-report. Prior myocardial infarction was defined as a physician-documented myocardial infarction preceding the index hospitalization. Obstructive coronary artery disease was defined angiographically as ≥50% luminal stenosis in at least one major epicardial coronary vessel. All patients underwent coronary angiography during the index hospitalization. DAPT consisted of aspirin plus a P2Y12 inhibitor. Clopidogrel-based DAPT represented the principal treatment strategy at the participating centers during 2022–2024, while ticagrelor was used selectively in patients considered to have higher ischemic risk. The exact patient-level distribution of P2Y12 inhibitors, adherence, and treatment duration was not retained as structured variables; therefore, the study cannot compare outcomes between clopidogrel and ticagrelor or estimate a treatment effect of escalation.

Troponin I was measured using the ARCHITECT STAT High Sensitive Troponin-I assay (Abbott Diagnostics, Abbott Park, IL, USA) on the ARCHITECT i2000 platform (Abbott Diagnostics, Abbott Park, IL, USA) using chemiluminescent microparticle immunoassay (CMIA) technology; the laboratory/manufacturer reference limit used locally was 0.04 ng/mL [7]. Samples with concentrations exceeding the initial analytical measurement range were remeasured after appropriate dilution according to laboratory procedures. Rechecking the source laboratory data confirmed that the very high values observed in a subset of patients, including concentrations ≥ 100 ng/mL, represented actual measured concentrations rather than data-coding artifacts. The archived dataset did not preserve whether the modelled value represented the first admission measurement or the peak in-hospital value. The WOE-derived threshold of ≥100 ng/mL was identified within this cohort and should therefore be regarded as a cohort-specific prognostic cut-off rather than a universal diagnostic threshold. Its prognostic applicability requires confirmation in an independent cohort.

Background lipid-lowering treatment during the study period consisted of high-intensity statin monotherapy as the routine secondary-prevention strategy. The Kazakhstan 2023 dyslipidemia protocol recommends adding ezetimibe when the LDL-C target is not achieved with maximally tolerated statin therapy and recommends an LDL-C target < 1.4 mmol/L for very-high-risk secondary prevention [8]. However, ezetimibe was not included in the state free outpatient formulary for ischemic heart disease during the study period, whereas atorvastatin was included [5], which limited routine access to combination lipid-lowering therapy in primary care. The median LDL-C values observed in this cohort (approximately 3.1 mmol/L) were substantially above the recommended target, consistent with incomplete LDL-C target attainment. Longitudinal LDL-C target achievement and individual adherence to lipid-lowering therapy were not available as structured variables.

### 2.3. Ethical Considerations

The study was conducted in accordance with the Declaration of Helsinki. Written informed consent was obtained from all participants before inclusion. The study protocol was reviewed and approved by the institutional ethics committee of Hodja Ahmed Yasawi International Kazakh-Turkish University (Protocol No. 53; 17 June 2024).

### 2.4. Statistical Analysis

Continuous variables are presented as median and interquartile range (Me [Q1; Q3]). Categorical variables are presented as frequencies and percentages. Between-group differences were assessed using the Mann–Whitney U test for continuous variables and the chi-square test or Fisher exact test for categorical variables, as appropriate.

Odds ratios (ORs) with 95% confidence intervals (CIs) were estimated using univariable logistic regression. A multivariable binomial logistic regression model was then fitted because the endpoint was binary and no time-to-event data were available.

Before exploratory score derivation, the cohort was randomly split in a 3:1 ratio into a derivation subset (75%; *n* = 1200) and a hold-out assessment subset (25%; *n* = 400). Continuous variables were transformed into binary predictors using Weight of Evidence (WOE) binning, and stepwise selection was used to derive a parsimonious bedside score. Only 36 deaths occurred in the full cohort. Evaluating multiple candidate predictors, applying data-driven cut-offs and dichotomization, performing stepwise selection, and using a single random hold-out split create substantial risks of overfitting, unstable coefficients, and optimistic performance estimates. The hold-out analysis was therefore used only as a preliminary out-of-sample assessment and is not presented as definitive internal validation. Retaining continuous predictors with flexible functional forms, penalized regression, and bootstrap- or repeated cross-validation would be methodologically preferable for subsequent model redevelopment and validation.

An exploratory point-based score was constructed from the fitted regression model by converting predictor weights into integer points. Discrimination and classification performance were summarized using receiver operating characteristic (ROC) analysis, area under the curve (AUC), sensitivity, specificity, and accuracy. The Youden index was used to identify a provisional classification threshold. These analyses were descriptive and hypothesis-generating because the event count was limited.

## 3. Results

### 3.1. Study Population

The final cohort included 1600 patients. Of these, 1564 patients survived and 36 patients died. Baseline demographic, clinical, laboratory, and instrumental characteristics are summarized in Table 1.

In the initial univariable analysis, prior myocardial infarction was the only variable with a statistically significant association with mortality (OR 2.09; 95% CI 1.06–4.09; *p* = 0.033). Patients with prior myocardial infarction therefore had more than a twofold increase in the odds of death compared with patients without prior myocardial infarction.

### 3.2. Exploratory Multivariable Modelling and Variable Selection

In the fitted multivariable model including all available variables, prior myocardial infarction remained statistically associated with mortality (OR 2.14; 95% CI 1.06–4.26; *p* = 0.030). Given the low number of events, this estimate should be interpreted as exploratory rather than as a stable independent prognostic effect. The full multivariable model is shown in Table 2.

Stepwise variable selection retained prior myocardial infarction, eGFR, smoking, BMI, and troponin I. Age was retained in the subsequent exploratory model because of its established clinical relevance despite borderline statistical significance (*p* = 0.072). Both the full and stepwise models were fitted in the derivation cohort (*n* = 1200). Rechecking the source dataset confirmed 36 deaths in the full analytical cohort of 1600 patients. However, the exact distribution of deaths between the original derivation and hold-out subsets could not be independently reconstructed because the original random-split assignment was not preserved in the archived analytic output. The marked narrowing of some confidence intervals after stepwise selection should not be interpreted as evidence of improved precision; with few events and data-driven model selection, model-selection-induced optimism and coefficient instability are plausible and require confirmation in a re-analysis using penalization and resampling.

### 3.3. Exploratory Score Derivation

For exploratory score derivation, continuous predictors were dichotomized using WOE binning. The resulting cut-off intervals are shown in Table 3. These cut-offs were then entered into the six-variable regression model with the categorical predictors retained during the preceding modelling stage. Because the cut-offs were data-derived, they should be regarded as provisional and require re-estimation in future studies.

The exploratory six-variable logistic regression model included age ≥ 57 years, eGFR < 45 mL/min/1.73 m^2^, BMI ≥ 25 kg/m^2^, troponin I ≥ 100, prior myocardial infarction, and current smoking. All variables were statistically significant within this fitted data-driven model (Table 4). Troponin I showed the strongest observed association with mortality (OR 6.57; 95% CI 2.48–19.33; *p* < 0.001), although the estimate is potentially unstable because of the limited event count and the cohort-specific threshold.

Integer points were assigned according to the relative strength of association in the fitted model. The resulting preliminary score reflects cumulative risk burden within this cohort and can be calculated using routinely available parameters (Table 5). The point weights should not be considered fixed or transportable until they are re-estimated and validated in larger datasets.

### 3.4. Exploratory Performance Assessment

For each patient in the derivation cohort, a total risk score was calculated. Within this fitted cohort, each additional point was associated with a 39% increase in the odds of mortality (OR 1.39; 95% CI 1.29–1.51; *p* < 0.001). This association represents apparent model performance and should not be interpreted as a transportable effect estimate.

Using the default probability threshold of 0.50 in the derivation model, mortality was predicted mainly at scores of 9–10 points. This threshold produced very high specificity but low sensitivity and was therefore not suitable as a practical screening threshold within this exploratory analysis.

**Figure 2:**
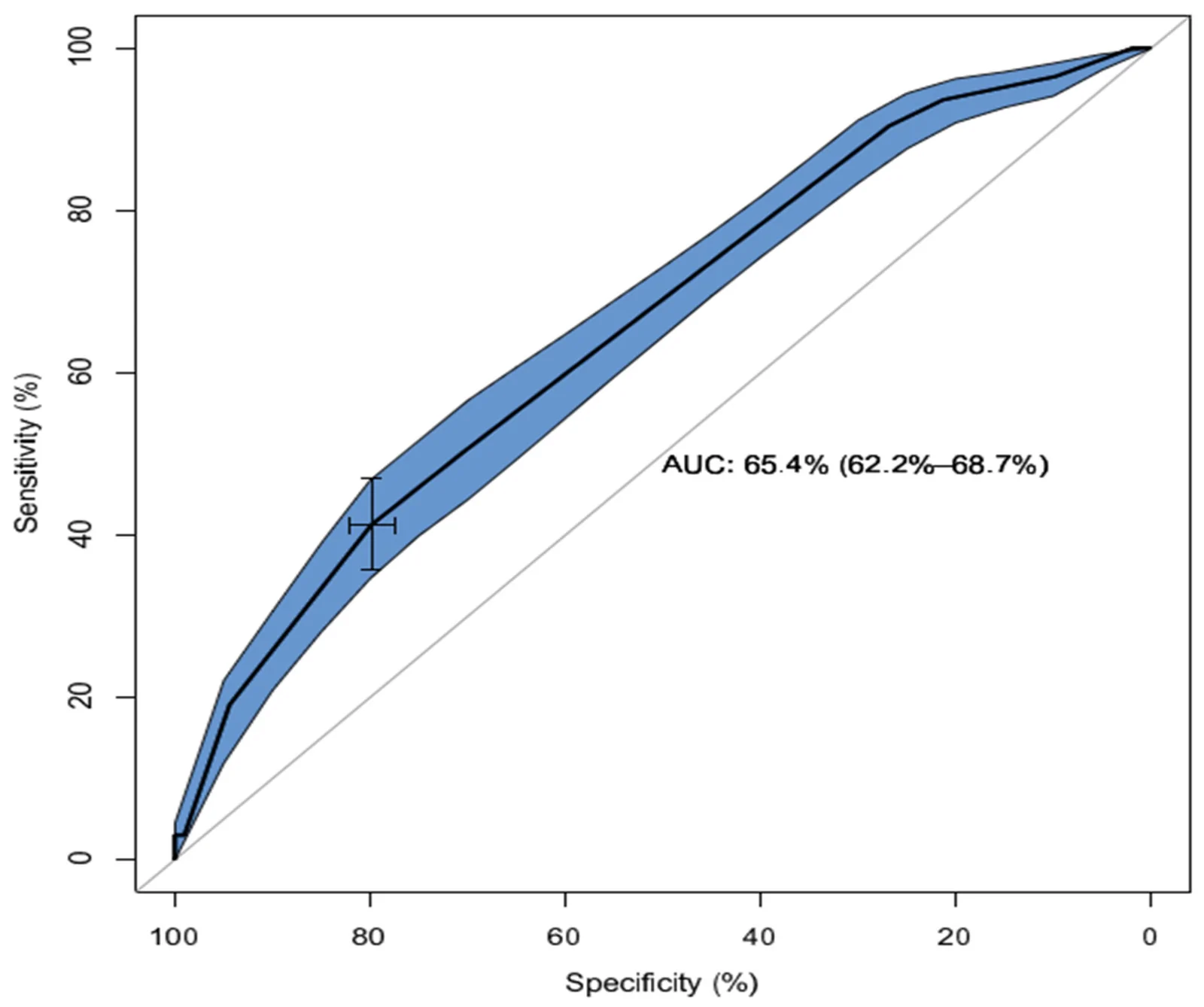
ROC curve for the exploratory clinical mortality score in the derivation cohort.

ROC analysis was used to identify a provisional decision threshold. The Youden-index threshold shifted the linear predictor to −0.96, corresponding to an estimated mortality probability of 28% and a total score of approximately 6 points. In the derivation cohort, this threshold yielded sensitivity of 0.41, specificity of 0.80, and overall accuracy of 0.72. The AUC was 0.654 (Figure 2). These values describe performance in the derivation data and are not evidence of definitive model validation.

The area under the ROC curve (AUC) was 65.4%. At the optimal cut-off determined by the Youden index (linear predictor = −0.96), the model demonstrated a sensitivity of 41.2% and a specificity of 79.7%. This cut-off corresponds to an estimated probability of death of approximately 28%; therefore, a linear predictor value of −0.96 was used as the provisional threshold for classifying patients as being at increased risk of in-hospital mortality.

In the hold-out assessment subset (*n* = 400), the score demonstrated sensitivity of 0.53, specificity of 0.70, accuracy of 0.70, and an AUC of 0.61. These values provide only a preliminary out-of-sample performance estimate and should not be interpreted as robust internal validation because of the low number of outcome events and the use of a single random split. A score around 6 points identified a higher-risk subgroup in this dataset, but the threshold remains provisional and should not be used to mandate platelet-reactivity testing or automatic antiplatelet escalation.

## 4. Discussion

In this study, we derived an exploratory clinical score for all-cause in-hospital mortality risk stratification in patients with coronary artery disease receiving dual antiplatelet therapy. The preliminary score included age, estimated glomerular filtration rate, body mass index, troponin I, prior myocardial infarction, and smoking status. These variables are routinely available and represent clinically plausible domains of early risk. However, because only 36 deaths occurred and the modelling strategy was data-driven, the present findings should be interpreted as hypothesis-generating rather than as evidence of a stable, validated prognostic instrument.

The present findings should be interpreted in the context of residual ischemic risk after percutaneous coronary intervention. High on-treatment platelet reactivity (HTPR) has been repeatedly associated with recurrent ischemic events in patients treated with clopidogrel after PCI. Previous studies have shown that increased platelet reactivity may contribute to recurrent myocardial infarction, repeat revascularization, and adverse outcomes after treatment for in-stent restenosis. Peri-procedural changes in platelet reactivity may also participate in myocardial injury through transient activation of hemostasis, localized thrombosis, and distal embolization [4,9]. Platelet reactivity is a dynamic biological process rather than a fixed laboratory characteristic. In acute ST-segment elevation myocardial infarction, platelet turnover and the presence of immature platelets may increase thrombotic potential and contribute to acute coronary thrombosis. Similar patterns of insufficient platelet inhibition have been described in other atherosclerotic conditions, including recurrent ischemic stroke and stable coronary artery disease, where high on-aspirin platelet reactivity has been associated with recurrent vascular events [10,11,12].

The prognostic relevance of platelet reactivity is also supported by studies linking high platelet reactivity with adverse cardiac remodeling and metabolic risk. In patients with acute myocardial infarction, elevated platelet reactivity has been associated with left ventricular remodeling and impaired functional recovery. Lipid-related and metabolic factors may further modulate platelet activity, suggesting that platelet reactivity reflects both pharmacological response and the patient’s broader thrombo-inflammatory profile [13,14].

Several studies have evaluated platelet function, P2Y12 inhibition, and clinical outcomes after PCI. High platelet reactivity after prasugrel loading has been associated with cardiovascular events in acute coronary syndrome, while hyporesponsiveness to aspirin or clopidogrel remains a clinically relevant problem after ACS. Long-term data after drug-eluting stent implantation also support the association between high platelet reactivity during clopidogrel therapy and unfavorable clinical outcomes [15,16,17].

The clinical effect of intensified antiplatelet therapy remains heterogeneous. High platelet reactivity affects outcomes after PCI, and pharmacodynamic studies suggest that more potent P2Y12 inhibition may reduce platelet reactivity more effectively than clopidogrel in selected patients. However, the clinical benefit of switching or intensifying therapy depends on baseline ischemic risk, bleeding risk, and patient selection. Reported predictors of high platelet reactivity include age, body mass index, diabetes mellitus, inflammatory markers, and fibrinogen level [18,19,20].

Interindividual variability in response to oral antiplatelet therapy is therefore central to post-PCI risk assessment. This variability may result from genetic polymorphisms, drug interactions, absorption differences, comorbid conditions, and baseline platelet reactivity. Platelet function assays, including vasodilator-stimulated phosphoprotein phosphorylation and platelet aggregation testing, may help identify patients with insufficient platelet inhibition. High on-treatment platelet reactivity has also been linked to stent thrombosis after coronary intervention [21,22,23].

At the same time, platelet reactivity should not be interpreted as an isolated determinant of prognosis. Platelet-fibrin clot strength, baseline thrombotic status, lesion complexity, inflammatory activity, and comorbidity burden may all modify post-PCI outcomes. This may explain why the prognostic value of HTPR differs across studies and why platelet reactivity testing alone has not produced uniform improvements in clinical outcomes. Large registry data after drug-eluting stent implantation support the association between platelet reactivity and ischemic events, but also emphasize the need for individualized risk assessment [24,25,26].

Current consensus statements support a selective rather than universal approach to platelet function and genetic testing after PCI. Such testing may be most useful when the expected clinical consequences of insufficient platelet inhibition are high. HTPR has also been studied in peripheral arterial disease, which supports the broader relevance of residual platelet activity across atherosclerotic vascular conditions. Standardized definitions and cut-off values remain important for translating platelet reactivity testing into routine practice [27,28,29].

The multifactorial nature of residual platelet activity is further supported by mechanistic and clinical studies. Platelet activation is influenced by intracellular regulatory pathways, platelet aggregation mechanisms, patient age, comorbidity profile, and background atherosclerotic burden. In elderly patients with coronary artery disease, clinical predictors of high-on-aspirin platelet reactivity have been identified, reinforcing the need to evaluate platelet reactivity within a broader clinical context [30,31].

Large-scale and disease-specific studies have further clarified this relationship. ADAPT-DES showed that platelet reactivity is related to stent thrombosis risk over time after drug-eluting stent implantation. Body mass index has also been associated with platelet reactivity during dual antiplatelet therapy with clopidogrel or ticagrelor. In peripheral arterial disease, available data indicate that optimal platelet function tests and clinically meaningful cut-off values still require further refinement [32,33,34].

The clinical positioning of the present exploratory score should be considered alongside established post-PCI risk stratification tools. PRECISE-DAPT and the DAPT score were developed primarily to inform bleeding/ischemic trade-offs and DAPT duration, whereas the present analysis explored whether routinely available variables could define a preliminary in-hospital mortality risk phenotype. The current score should not be viewed as a validated alternative or established complement to these instruments. A direct head-to-head comparison, model redevelopment using event-efficient methods, and external validation are required before incremental clinical value can be claimed. Contemporary management after PCI emphasizes individualized selection of the P2Y12 inhibitor according to ischemic and bleeding risk [6,35]. In the participating Kazakhstan centers, where clopidogrel-based DAPT was the principal strategy, identification of a clinically high-risk patient may reasonably prompt reassessment of the antiplatelet regimen and consideration of ticagrelor when ischemic risk is high and bleeding risk acceptable. This implication derives from guideline-based clinical assessment, not from a validated treatment-selection property of the exploratory score.

Residual lipid risk is also relevant to interpretation of the cohort. High-intensity statin monotherapy was the routine lipid-lowering strategy, while routine access to ezetimibe through primary-care reimbursement was limited during the study period [5]. This treatment context differed from the stepwise combination strategy recommended in the Kazakhstan 2023 dyslipidemia protocol, which advises ezetimibe when LDL-C remains above target despite maximally tolerated statin therapy [8]. Median LDL-C concentrations around 3.1 mmol/L in the cohort were well above the recommended <1.4 mmol/L target for very-high-risk secondary prevention. Thus, incomplete lipid control represents an important component of residual cardiovascular risk and may have contributed to the overall risk profile, although the study was not designed to quantify the effect of lipid-lowering treatment on mortality.

The potential clinical value of the exploratory score is that it uses variables available before specialized platelet testing is considered. Troponin I, renal function, body mass index, prior myocardial infarction, age, and smoking status are clinically plausible markers of risk. The markedly elevated troponin I concentrations observed in a subset of patients may be explained, at least in part, by relatively late presentation after the index ischemic event and more extensive myocardial injury in some patients. However, these factors were not formally evaluated in the present analysis, and this explanation should be regarded as clinically plausible rather than demonstrated. At the present stage, the score should be regarded as a framework for future prospective investigation rather than as a bedside rule. Its predictor weights, cut-offs, calibration, and incremental value require re-estimation and validation in larger cohorts with more outcome events.

This interpretation should remain cautious. Platelet function was not measured, and the score predicts all-cause in-hospital mortality rather than high on-treatment platelet reactivity. No direct association between the score and platelet reactivity can therefore be inferred. Likewise, because patient-level P2Y12 treatment allocation was not available, the study cannot demonstrate that switching from clopidogrel to ticagrelor improves outcomes in patients with a high score. The appropriate interpretation is a risk-enrichment hypothesis: patients displaying the identified high-risk features may warrant structured reassessment of ischemic risk, bleeding risk, adherence, lipid control, and antiplatelet strategy on established clinical grounds. Platelet function or genetic testing, where available, and escalation to ticagrelor should not be triggered by the score alone.

Overall, this study provides a preliminary framework for exploratory in-hospital mortality risk stratification in patients with coronary artery disease receiving DAPT. The modest discrimination, low number of deaths, and data-driven modelling workflow preclude use as a stand-alone prognostic or treatment-selection instrument. Future work should redevelop the model using penalized or otherwise event-efficient methods with resampling-based internal validation, followed by prospective multicenter external validation incorporating detailed P2Y12 exposure, platelet function testing, contemporary lipid-lowering therapy, and procedural PCI variables.

## 5. Conclusions

This exploratory study derived a preliminary clinical score for all-cause in-hospital mortality risk stratification in patients with coronary artery disease receiving dual antiplatelet therapy after percutaneous coronary intervention. The score contains six routinely available variables: age, estimated glomerular filtration rate, body mass index, troponin I, prior myocardial infarction, and smoking status. A score of approximately 6 points identified a higher-risk subgroup within this dataset, but this threshold is provisional.

The observed discrimination was modest, and the single hold-out assessment does not constitute definitive internal validation. The score should therefore be viewed as a hypothesis-generating risk-enrichment framework. In the clopidogrel-dominant practice setting studied, the identified clinical risk pattern may support broader reassessment of secondary prevention, including consideration of whether ticagrelor is appropriate after an independent evaluation of ischemic and bleeding risk. The present study does not establish a causal benefit of ticagrelor or a direct relationship between the score and platelet reactivity.

The score is not ready for routine clinical decision-making and should not substitute for validated risk tools, platelet function testing, or individualized clinical judgment. Model redevelopment and resampling-based internal validation in a dataset with an adequate number of events, followed by independent external validation, are required before clinical implementation can be considered.

## 6. Limitations

This study has several limitations. First, the retrospective design may have introduced selection bias and limits causal interpretation. Second, the analysis was based on a single regional cohort, which may restrict generalizability to other populations and healthcare systems. Third, the random 3:1 hold-out assessment was based on the same source cohort and, especially with few events, does not constitute robust internal or external validation. The resulting performance estimates should therefore be regarded as preliminary.

Fourth, time-to-event data were not available, so Kaplan–Meier analysis and Cox proportional hazards modelling could not be performed. Fifth, platelet function testing, CYP2C19 genotype, drug interactions, individual adherence, exact DAPT duration, and patient-level P2Y12 agent assignment were not available as structured variables. Although clopidogrel-based DAPT was the principal institutional strategy, the study cannot compare clopidogrel with ticagrelor or infer that treatment escalation would reduce mortality. Sixth, only 36 deaths occurred. This is the central methodological limitation of the score: the low events-per-predictor ratio, data-driven variable selection, dichotomization, and score construction create substantial risk of overfitting, coefficient instability, exaggerated apparent associations, and optimistic performance estimates. Accordingly, all score-related findings are exploratory.

Seventh, coronary anatomical complexity (e.g., multivessel disease, lesion complexity, bifurcation lesions, chronic total occlusions, and calcification) and detailed procedural characteristics (number and total length of stents, intravascular imaging, post-dilatation, and procedural/angiographic success) were not captured and may have limited predictive performance. Eighth, high-intensity statin monotherapy was the routine lipid-lowering strategy; however, longitudinal adherence and LDL-C target attainment were not systematically recorded. Ezetimibe was recommended by the national dyslipidemia protocol when statin therapy was insufficient but was not included in the state free outpatient formulary for ischemic heart disease during the study period, limiting routine access to combination therapy [33,34]. Ninth, calibration plots, calibration intercept and slope, Brier score, confidence intervals for performance measures, and decision-curve analysis were not available from the archived analysis. These measures, together with bootstrap or repeated cross-validation and preferably penalized modelling, are required before the score can be considered adequately internally validated. Tenth, although the very high troponin I concentrations were confirmed after appropriate dilution, the ≥100 ng/mL threshold was selected using WOE binning within this cohort and may not be fully transportable to other populations or analytical platforms; independent validation is required. Finally, rechecking the source dataset confirmed 36 deaths in the full cohort. Because the original random-split assignment was not preserved in the archived analytic output, the exact split-specific event counts could not be independently reconstructed, which further limits interpretation of the hold-out assessment.

## Figures and Tables

**Table 1:** Baseline characteristics of surviving and deceased patients.

	Survived N = 1564	Died N = 36	OR	*p* Ratio	*p* Overall	N
Age	66.0 [59.0; 73.0]	64.0 [61.0; 73.0]	1.01 [0.98; 1.05]	0.490	0.744	1599
Sex:					0.459	1600
Female	706 (45.1%)	19 (52.8%)	Reference	Reference		
Male	858 (54.9%)	17 (47.2%)	0.74 [0.37; 1.44]	0.369		
eGFR	76.0 [63.0; 89.0]	74.0 [62.0; 84.8]	0.99 [0.97; 1.01]	0.279	0.510	1587
BMI	27.7 [25.0; 31.0]	28.5 [25.8; 30.4]	1.01 [0.95; 1.08]	0.658	0.595	1600
CRP	3.30 [2.20; 6.18]	3.30 [2.04; 4.45]	0.97 [0.92; 1.03]	0.298	0.509	1580
Total cholesterol	4.92 [4.12; 5.78]	5.11 [3.73; 6.00]	1.00 [0.99; 1.01]	0.972	0.925	1600
LDL-C	3.10 [2.35; 3.86]	3.21 [2.18; 3.81]	1.00 [0.74; 1.35]	0.994	0.959	1599
Triglycerides	1.30 [1.11; 1.77]	1.30 [1.16; 1.50]	0.80 [0.52; 1.21]	0.286	0.597	1600
Fibrinogen	3.25 [2.80; 3.85]	3.54 [2.94; 4.18]	1.09 [0.78; 1.52]	0.612	0.339	1599
Type 2 diabetes mellitus:					0.783	1600
No	1183 (75.6%)	26 (72.2%)	Reference	Reference		
Yes	381 (24.4%)	10 (27.8%)	1.21 [0.55; 2.46]	0.627		
Prediabetes:					0.624	1598
No	1515 (97.0%)	36 (100%)	Reference	Reference		
Yes	47 (3.01%)	0 (0.00%)	Not estimable	Not estimable		
COVID-19:					0.134	1600
No	1255 (80.2%)	33 (91.7%)	Reference	Reference		
Yes	309 (19.8%)	3 (8.33%)	0.39 [0.09; 1.09]	0.076		
COPD:					1.000	1600
No	1511 (96.6%)	35 (97.2%)	Reference	Reference		
Yes	53 (3.39%)	1 (2.78%)	0.93 [0.04; 4.38]	0.942		
Coronary vessel disease:					0.881	1600
Non-obstructive	737 (47.1%)	16 (44.4%)	Reference	Reference		
Obstructive	827 (52.9%)	20 (55.6%)	1.11 [0.57; 2.20]	0.756		
D-dimer	200 [110; 440]	200 [100; 420]	1.00 [1.00; 1.00]	0.902	0.998	1587
Troponin I	0.00 [0.00; 0.01]	0.00 [0.00; 0.01]	1.01 [0.99; 1.02]	0.520	0.275	1597
Ejection fraction	58.0 [51.0; 64.0]	59.5 [51.8; 63.2]	1.01 [0.98; 1.05]	0.415	0.619	1600
Smoking:					0.723	1600
No	1275 (81.5%)	28 (77.8%)	Reference	Reference		
Yes	289 (18.5%)	8 (22.2%)	1.28 [0.53; 2.72]	0.558		
Hemoglobin	140 [130; 151]	136 [128; 148]	1.00 [0.98; 1.01]	0.694	0.511	1598
Prior myocardial infarction:					0.041	1599
No	1095 (70.1%)	19 (52.8%)	Reference	Reference		
Yes	468 (29.9%)	17 (47.2%)	2.09 [1.06;4.09]	0.033		

Abbreviations: eGFR, estimated glomerular filtration rate, CRP, C-reactive protein, BMI, body mass index, LDL-C, low-density lipoprotein cholesterol, COPD, chronic obstructive pulmonary disease.

**Table 2:** Exploratory multivariable logistic regression model for in-hospital mortality in the derivation cohort.

Predictors	OR	95% CI	*p*
Age	1.01	0.97–1.05	0.725
Sex: male	0.81	0.35–1.85	0.627
eGFR	0.99	0.97–1.01	0.472
BMI	1.03	0.96–1.10	0.428
CRP	0.96	0.88–1.00	0.181
Total cholesterol	1.01	0.75–1.49	0.949
LDL-C	1.00	1.00–1.00	0.995
Triglycerides	0.75	0.43–0.99	0.235
Fibrinogen	1.11	0.74–1.64	0.597
Type 2 diabetes mellitus: Yes	1.35	0.59–2.89	0.450
COVID-19: Yes	0.36	0.09–1.04	0.098
COPD: Yes	0.69	0.04–3.51	0.725
Coronary vessel disease: obstructive	1.08	0.54–2.15	0.835
D-dimer	1.00	1.00–1.00	0.370
Troponin I	1.01	0.98–1.03	0.285
Ejection fraction	1.02	0.99–1.05	0.293
Smoking: Yes	1.86	0.71–4.56	0.188
Hemoglobin	1.00	0.98–1.02	0.692
Prior myocardial infarction: Yes	2.14	1.06–4.26	0.030

Abbreviations: OR, odds ratio; CI, confidence interval; eGFR, estimated glomerular filtration rate; BMI, body mass index; CRP, C-reactive protein; LDL-C, low-density lipoprotein cholesterol; COPD, chronic obstructive pulmonary disease.

**Table 3:** Cut-off intervals of continuous variables according to the Weight of Evidence binning algorithm.

Variable	Low Risk	High Risk
Age	<57 years	≥57 years
eGFR	≥45	<45
BMI	<25	≥25
Troponin I, ng/mL	<100	≥100

**Table 4:** Six-variable logistic regression model used for exploratory mortality-score derivation in the derivation cohort.

Predictors	OR	95% CI	*p*
Age ≥ 57 years	2.81	1.95–4.14	<0.001
eGFR < 45	2.59	1.65–4.01	<0.001
BMI ≥ 25	1.90	1.37–2.69	<0.001
Troponin I ≥ 100 ng/mL	6.57	2.48–19.33	<0.001
Prior myocardial infarction	2.31	1.83–2.91	<0.001
Smoking	1.36	1.03–1.80	0.030

Abbreviations: OR, odds ratio; CI, confidence interval; eGFR, estimated glomerular filtration rate; BMI, body mass index.

**Table 5:** Exploratory clinical score for all-cause in-hospital mortality risk stratification.

Variable	High Risk	Score
Age	≥57 years	3
eGFR	<45 mL/min/1.73 m^2^	3
BMI	≥25 kg/m^2^	2
Troponin I ≥ 100 ng/mL	≥100	7
Prior myocardial infarction	Presence of prior MI	2
Smoking	Current smoker	1

Note: Troponin I was measured with the Abbott ARCHITECT STAT High Sensitive Troponin-I assay on the ARCHITECT i2000 platform. Values exceeding the initial analytical measurement range were confirmed after appropriate dilution. The score cut-off of ≥100 ng/mL was derived from the present cohort using WOE binning and is not presented as a manufacturer-defined diagnostic concentration or a universally applicable clinical threshold.

## Data Availability

The data supporting the findings of this study are available from the corresponding author upon reasonable request, subject to institutional and ethical restrictions on patient-level medical record data.

## Abbreviations

The following abbreviations are used in this manuscript:

Abbreviation	Definition
ACS	acute coronary syndrome
AUC	area under the curve
BMI	body mass index
CAD	coronary artery disease
CI	confidence interval
COPD	chronic obstructive pulmonary disease
CRP	C-reactive protein
DAPT	dual antiplatelet therapy
eGFR	estimated glomerular filtration rate
HTPR	high on-treatment platelet reactivity
LDL-C	low-density lipoprotein cholesterol
MI	myocardial infarction
OR	odds ratio
PCI	percutaneous coronary intervention
ROC	receiver operating characteristic
WOE	Weight of Evidence
